# Investigating Synthetic Dolomite Mixtures and Phase Adjustment Parameters in Electrochemical Cement Precursor Production

**DOI:** 10.1002/cphc.202500809

**Published:** 2026-04-22

**Authors:** Anthony R. Ramuglia, Phong V. Ly, Sophie Hurst‐Fraunhofer, Julius Scheel, Kelly Henze, Thomas Köberle, Volodymyr Bon, Stefan Kaskel, Viktor Mechtcherine, Thomas Matschei, Marco Liebscher, Inez M. Weidinger

**Affiliations:** ^1^ Chair of Electrochemistry, Faculty of Chemistry and Food Chemistry Technische Universität Dresden Dresden Germany; ^2^ Institute of Construction Materials Technische Universität Dresden Dresden Germany; ^3^ Chair of Physical Chemistry, Faculty of Chemistry and Food Chemistry Technische Universität Dresden Dresden Germany; ^4^ Institute of Building Materials Research RWTH Aachen University Aachen Germany

**Keywords:** cement chemistry, cement precursors, electrolysis, sustainable cement

## Abstract

The precursor material of Portland cement (PC) colloquially referred to as clinker is produced primarily via calcination of limestone (CaCO_3_). Magnesia‐based cements can similarly be formed via calcination of magnesite (MgCO_3_) among other routes. Dolomitic minerals typically contain a mixture of these carbonate mineral phases. The current study monitors and explores the electrochemical transformation of CaCO_3_ and MgCO_3_ synthetic mixtures approximating dolomite and investigates their applicability in forming cementitious phases. The transformation of such mixtures indicates that Mg‐ and Ca‐based electrolysis products, namely Ca(OH)_2_ and Mg(OH)_2_, respectively, display different precipitation parameters. Mg(OH)_2_ precipitates more rapidly in the form of flower‐shaped micro‐aggregates, while Ca(OH)_2_ forms slower in comparison, precipitating in larger hexagonal prismatic structures. These findings point toward possible mixture separation techniques for PC and magnesia‐based cements.

## Graphical abstract

The electrochemical dissolution of synthetic dolomite mixtures and reconstitution into hydroxide precursor phases suitable for Portland cement and Mg‐based cements is investigated. Adjustments of the experimental parameters during electrolysis allow for adjustments in product phase distribution, shedding insight into mixture separation techniques in electrochemical cement precursor production.

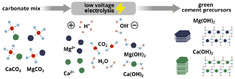

## 1 Introduction

Cement is the most ubiquitous man‐made material in the world [1]. It is the major active component in concrete, which is the foundation of bridges, roads, dams, and a plethora of other essential infrastructure. However, due to its prevalence and utility, cement production alone constitutes approximately 8% of all anthropomorphic CO_2_ produced globally per year [2]. Cement, or more commonly Portland cement (PC), is an encompassing term, which typically refers to a mixture of calcium oxide mineral phases consisting of silicates, aluminates, and ferrites. These calcium‐based phases are referred to as cement clinker and depending on the region and source material the mixture composition of these clinker phases may differ. Predominately, these clinker phases are polymorphs of calcium silicate, namely alite (Ca_3_SiO_5_) and belite (Ca_2_SiO_4_), with alite compromising approximately 60–70% of typical PC [3]. These polymorph phases themselves are produced via an energy intensive clinkering process. Initially, limestone (calcium carbonate) CaCO_3_ is mixed with silicate‐ and aluminate‐based minerals and heated in a precalciner at approximately 900°C, resulting in the calcination of CaCO_3_ into the calcium oxide (CaO) and the release of CO_2_. The resultant CaO silicate and aluminate mix is then heated in a rotary kiln at extreme temperatures up to 1450°C. These temperatures promote the solid state formation of alite and belite phases and result in solid cement clinker nodules. Magnesium‐based cements differ from conventional PC in their phase composition, however, the formation of Mg‐based cements can be generated from magnesium carbonate (magnesite) MgCO_3_, using similar heating techniques albeit at lower temperatures.

Importantly, these thermal processes required for the decarbonisation of minerals and the formation of clinker are energy intensive and are currently conducted through the combustion of fossil fuels or coal, substantially exacerbating greenhouse gas emissions through the manufacturing process [4]. Consequently, electrochemistry has been proposed as a more sustainable methodological approach to PC clinker precursor production [5, 6, 7, 8]. The core principle of this method involves utilizing an electrolyzer to dissolve CaCO_3_ and transform it into calcium hydroxide (Ca(OH)_2_) while off‐gassing pure CO_2_. This electrochemical approach alleviates the need for CaCO_3_ calcination into CaO, circumventing the formation of the high energy CaO precursor material (ΔH^*f*^^°^ = −634.9 kJ/mol for CaO vs Δ*H* ^*f*^^°^ = −985.2 kJ/mol for Ca(OH)_2_) [5, 6, 7, 9, 10]. The generated Ca(OH)_2_ is a precursor to clinker, producing phases identical to clinker from CaO when used as a source material [5]. The electrochemical transformation of CaCO_3_ is facilitated by the electrolysis of water, wherein water is oxidized at the anode, forming H^+^ and O_2,_ and reduced at the cathode, forming OH^−^ and H_2_, as shown in Equation (1) and (2), respectively.

(1) $$2H_{2}O\;\to \;O_{2}+4H^{+}+4e^{-}$$

(2) $$4H_{2}O+4e^{-}\;\to \;2H_{2}+4{\mathrm{OH}}^{-}$$

This electrochemical process can be equivocal in energy consumption as the conventional method [5] yet allow for the use of renewable sources to power the electrolyzer, i.e., wind and solar, perpetuating a green energy process. Congruently, other electrochemical systems have also eluded to the utility of Ca(OH)_2_ and Mg(OH)_2_ for use in carbon recycling and the formation of other cementitious precursor at scale [11, 12, 13, 14]. Although PC‐ and Mg‐based cements are viable cementitious materials, utilizing mixtures of calcium‐ and magnesium‐based minerals for the formation of such materials can be problematic. Magnesium oxide (MgO) can improve the stability when used as a small additive in PC, (∼1 wt %) [15], however, higher concentrations (>5%) result in the formation of the Mg‐based mineral brucite resulting in dimensional stability problems during delayed hydration [16]. Moreover, at atmospheric pressure, high temperatures required for alite formation would vaporize Mg within the mixture, causing deleterious effects to equipment. Similarly, concentrations of Ca^2+^ ions (>5%) are detrimental in Mg‐based cements, which can destabilize Mg‐based cementitious phases [17]. Therefore, in a practical sense regarding cement manufacturing, carbonate minerals mixtures or minerals such as dolomite (CaMg(CO_3_)_2_), which contain mixtures of both alkaline‐earth metal cations (Ca^2+^ in slight surplus) [18], are typically neither suitable minerals for traditional PC nor Mg‐based cements.

In this report, we investigate on such synthetic mixtures containing CaCO_3_ and MgCO_3_, their transformation electrochemically into the corresponding hydroxide analogs, and the applicability in forming a cementitious phase. Subsequently, product separation of Ca(OH)_2_ and Mg(OH)_2_ is investigated, which eludes to potential applications in clinker precursor production for both traditional PC‐ and Mg‐based cements within the broader context of CO_2_ abatement in cement manufacturing.

## 2 Results and Discussion

### 2.1 Monitoring Electrochemical Dissolution

**FIGURE 1:**
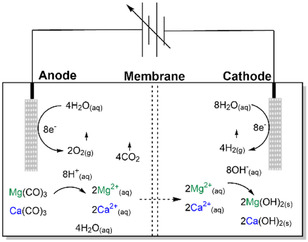
Schematic diagram of the electrochemical “H” cell where water electrolysis results in the transformation of the carbonate minerals.

A laboratory scale electrolyzer was constructed using a two‐compartment electrochemical cell in 0.5 M KNO_3,_ where the anode and cathode, both consisting of platinum on titanium mesh, were separated by a Nafion 115 cation exchange membrane. A synthetic substrate mixture of 1.00:0.84 mol Ca(CO)_3_:Mg(CO)_3_ was added to the anodic compartment of the electrolyzer to facilitate dissolution. For more details regarding the experimental setup the reader is referred to the experimental section within the SI. A schematic illustration of the two compartment electrolyzer is depicted in Figure 1. Linear sweep voltammetry (LSV) was conducted and indicate the no early onset nor other electrochemical phenomena are observed in the presence of substrate, which suggests no additional oxidative side reactions of the substrate occur within the potential window (Figure S1). The dissolution of the substrate is therefore purely a result of interaction with the H^+^ ions generated via water oxidation.

Within the anodic compartment, the CaCO_3_ and MgCO_3_ compounds are dissolved in the proton‐rich environment generated by the anode to form Ca^2+^, Mg^2+^, CO_3_^2−^_(aq)_ (carbonate equilibrium omitted for brevity), CO_2(g),_ and water. The resulting alkali‐earth ions diffuse through the Nafion membrane toward the negatively charged cathode where they interact with hydroxide ions to form their respective hydroxide products. The overall reactions for the transformation of the carbonate minerals are as follows, where M denotes either Ca or Mg

(3) $${\mathrm{MCO}}_{3}+2H^{+}\;\to \;M^{2+}+{\mathrm{CO}}_{2}+H_{2}O$$

(4) $$M^{2+}+2{\mathrm{OH}}^{-}\;\to \;M{\left(\mathrm{OH}\right)}_{2}$$

**FIGURE 2:**
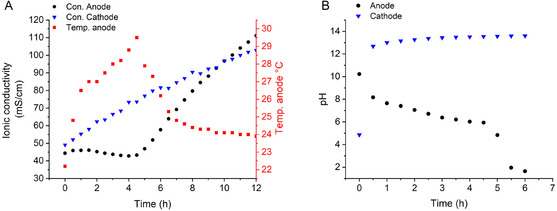
(A) Ionic conductivity and temperature (B) pH measurements of the reaction medium in the anodic compartment (black dots and red squares) and the cathodic compartment (blue triangles) during chronopotentiometry conducted at 100 mA at approximately 2.0 V.

The anodic compartment containing the carbonate mixture was stirred and pulsed chronopotentiometry was conducted at 100 mA with a cell voltage of approximately 2.0 V, adequate enough to overcome the thermodynamic barrier of water oxidation on Pt [19, 20] resulting in a power output of ∼0.20 W (Figure S2). The ionic conductivity at the anode and cathode directly adjacent to the electrodes was monitored over time and overlaid with temperature measurements at the anode, depicted in Figure 2A. The pH of both compartments was also monitored over time and is depicted in Figure 2B.

From the conductivity measurements, it is apparent that the ionic conductivity at the anode (black circles) remains relatively stagnant on the electrolysis time scale until approximately 4.5 h, after which time a steady increase is observed. Comparatively, the ionic conductivity at the cathode (blue triangles) increases continually from the onset. This discrepancy can be explained as protons generated at the anode interact directly with the substrate (CaCO_3_ and MgCO_3_) and dissolve the carbonate species. The resultant Ca^2+^ and Mg^2+^ ions then migrate toward the cathode, resulting in an ion gradient within the cell. The conductivity at the cathode continually increases, as OH^−^ is likely produced at a rate kinetically faster than the dissolution carbonate species at room temperature [21, 22]. The ionic conductivity at the anode however surpasses that of the cathode after 10 h due to the higher molar ionic conductivity of H^+^ ions (34.9 mS·m^2^·mol^−1^) compared to OH^−^ ions (19.8 mS·m^2^·mol^−1^) [23]. The temperature within the anodic compartment is also observed to increase over time, reaching a maximum of 29.5°C after 4.5 h (red squares) due to carbonate dissolution which is exothermic in acidic media. This is significant as this increase in temperature can have implications in energy production or storage within larger systems. The change in temperature and ionic conductivity after 4.5 h at the anode is also reflected in the corresponding pH measurements (Figure 2B). At the beginning of electrolysis, the pH of the anode (black circles) is basic (10.2), owing to the basicity of the carbonate species. However, over the course of the electrolysis, the pH at the anode gradually decreases until 4.5 h, after which time a more significant drop off is observed to low pH. The conductivity, pH, and temperature measurements taken together indicate the substrate has completely or nearly completely dissolved under acidic conditions after this initial 4.5 h at ∼0.20 W. Assuming the dissolution had completed after 4.5 h, the dissolution rate of the mixture can be approximated as 1.21·10^4^ mol/mWh. However, as the dissolute of each mole of carbonate substrate requires 2 moles of electrons, the columbic efficiency would exceed 100%. Upon further inspection, small amounts of substrate had collected at the membrane partition, suggesting the dissolution of the materials is effective in the immediate vicinity of the electrode in the presence of stirring, however shortcomings in cell design hinder complete substrate dissolution (Figure S3). Nevertheless, the faradaic efficiency (FE) of the governing electrochemical water splitting calculated through measuring the H_2_ evolution at the cathode was found to be approximately 85.7%, suggesting the system operates at relative high current efficiencies as electrolysis is used solely to oxidize and reduce water (Table S1 and S2, Figure S4).

**FIGURE 3:**
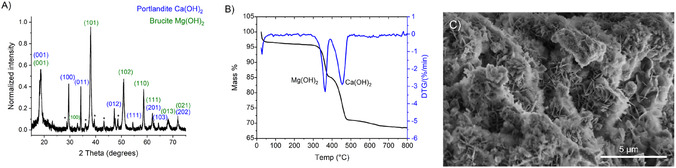
(A) XRD diffractogram (B) TGA/DTG curve (C) SEM image of the hydroxide product mixture collected within the cathodic compartment after chronopotentiometry conducted at 100 mA at approximately 2.0 V.

### 2.2 Hydroxide Product Characterization

The resulting precipitate which had accumulated within the cathodic compartment after substrate dissolution was collected and analyzed via powder X‐ray diffraction (XRD), thermogravimetric analysis (TGA), and scanning electron microscopy (SEM) and is displayed in Figure 3A–C.

As evident from the XRD analysis, the resulting product obtained is a mixture of brucite (Mg(OH)_2_) ICSD(79031) $\overset{―}{3}$ and portlandite (Ca(OH)_2_) ICSD(15471), both crystallize in a trigonal space group *P* $\overset{―}{3}$ *m*1. Experimentally measured   XRD diffractogr agree well to the literature values for the corresponding hydroxide species [24]. The asterisks on the XRD indicate reflections, which correspond to calcite (CaCO_3_) ICSD(52151), suggesting some degree of carbonation of the Ca(OH)_2_ from the atmosphere during handling. From the TGA measurements, the calculated change in mass % for Mg(OH)_2_ and Ca(OH)_2_ is 10.6% and 14.2% respectively. This corresponds to a ratio of 48.8 mol% Mg and 51.2 mol% Ca in good agreement with energy dispersive X‐ray spectroscopy (EDX) of the surface with 24.8 mol% of Ca and 24.9 mol% Mg (Table S3). SEM images of the product depict large substructures consisting of flower‐shaped surface aggregates spanning approximately 2–3 μm. These findings indicate the product exists as a near equal mixture of both hydroxide phases. However, such 1:1 mixtures containing both alkali‐earth ions (Ca^2+^ and Mg^2+^) are typically unsuitable for PC‐ or Mg‐based cements due to the high concentration of both ions. The resultant product mixture was nevertheless analyzed for its suitability in forming cementitious phases after heat treatment. Heating the product mixture at 650°C for 3 h in air results in the decomposition of both Ca(OH)_2_ and Mg(OH)_2_ mineral phases and calcination into their corresponding oxide phases, CaO and MgO (Figure S5) [25, 26]. The thermodynamic decomposition of Ca(OH)_2_ and Mg(OH)_2_ to their respective oxide species occurs at significantly lower temperatures than their carbonate analogs, highlighting the utility of the electrochemical process [5, 27]. A subsequent sample of the electrochemically generated product mixture containing Ca(OH)_2_ and Mg(OH)_2_ was combined with amorphous SiO_2_ in a 3:1 molar ratio and heated at 900°C for 5 h to promote the formation of cementitious phases. Unfortunately, heat treatment of this mixture resulted primarily in the corresponding oxide products, with powder XRD reflections depicting only trace amount of the phase reflections that may be attributed to the cementitious phase belite, although SEM images of the product suggest heating has also resulted in some amount of a glass‐like phase (Figure S6 and S7).

### 2.3 Adjusting Product Distribution via Electrolysis Time

**FIGURE 4:**
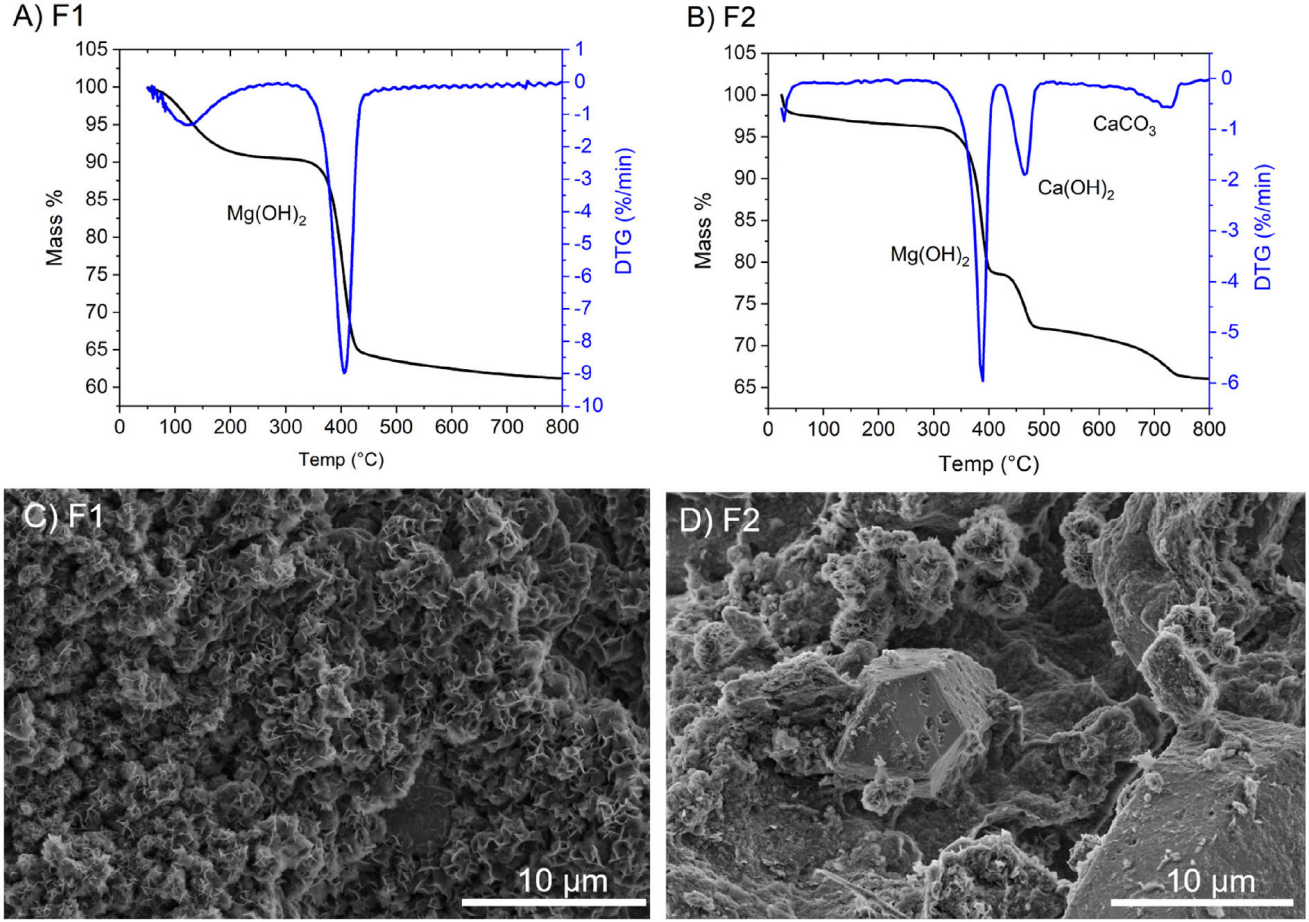
TGA/DTG curve of (A) Precipitate fraction F1 collected after 2.5 hr (B) precipitate fraction FT collected after 24 hr. SEM images of (C) precipitate fraction F2 collected after 2.5 hr (D) precipitate fraction F2 collected after 24 hr.

Alite, the predominate cementitious phase in PC, requires temperatures within the range of 1400–1500°C [5]. However, heating such mixtures of Ca(OH)_2_, Mg(OH)_2_ to these temperatures results in a glass material, presumably diopside, rather than cement clinker (Figure S8) [28]. Ultimately, in order to procure alite for Ca‐based cements, such mixtures require separation to reduce the Mg^2+^ concentration. Therefore, additional experiments were performed to find possible adjustments to the product composition. Chronopotentiometric electrolysis was conducted on the system as before (100 mA at approximately 2.0 V, ∼0.20 W); however, electrolysis was halted after 2.5 h, and the cathodic precipitate was filtered, collected (Figure S9) and abbreviated F1. Electrolysis was restarted in the presence of fresh electrolyte and after 24 h a second precipitate fraction was subsequently filtered and collected and abbreviated F2. Both fractions (F1 and F2) were analyzed via TGA, SEM, and EDX spectroscopy, with TGA and SEM measurements shown in Figure 4A–D. The TGA curve of the initial precipitate fraction (F1) indicates it is composed primarily of Mg(OH)_2_. EDX analysis underlines these findings quantitatively and indicates fraction F1 is  composed mainly of Mg (2.3 mol% Ca vs. 47.7 mol% Mg Table S4). TGA analysis of the second precipitate fraction (F2) indicates the precipiate contains three products, Mg(OH)_2_, Ca(OH)_2,_ and CaCO_3_, with CaCO_3_ a result of carbonation of Ca(OH)_2_ from handling. The mass percent loss from the curves of the three phases was calculated be 17.63%, 6.56%, and 4.31%, respectively. This corresponds to a mol% of 30.3% Ca and 69.7% Mg. Interestingly, EDX analysis of the suggest Mg is present in larger percentages on the surface aggregates (10.1 mol% Ca vs. 38.0 mol% Mg, Table S5). Nevertheless, these differences in mol% of the collected phases (F1 and F2) correlate to differences in the surface morphologies of the collected products. SEM images shown in Figure 4 of the first precipitate fraction, F1 (A), depict a microstructure morphology with flower‐shaped surface aggregates, indicating these surface structures are brucite (Mg(OH)_2_) precipitates. The second hydroxide product fraction, F2 (B), displays a morphological structure comprised of larger hexagonal prismatic crystals, indicative of portlandite (Ca(OH)_2_) dispersed within these flower‐shaped aggregates of brucite. XRD analysis confirms the formation of these hydroxide phases within the collected products (Figure S10).

### 2.4 Adjusting Product Distribution via Mixture Agitation

**FIGURE 5:**
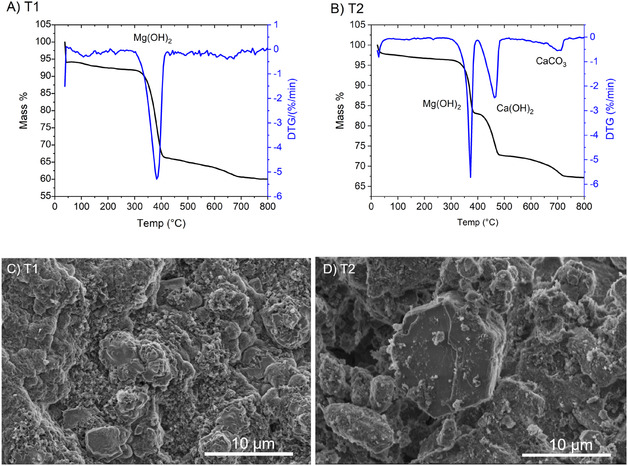
TGA/DTG curve of (A) precipitate fraction T1 collected after 20 h in the absence of magnetic stirring (B) precipitate fraction FT collected after 72 h. SEM images of (C) precipitate fraction T1 collected after 20 h in the absence of magnetic stirring (D) precipitate fraction T2 collected after 72 h.

The effect of mixture agitation on the system to impact product distribution was also investigated. Further electrolysis experiments were performed as before (chronopotentiometry at 100 mA at approximately 2.0 V); however, the mixture within the anodic compartment was not magnetically stirred. In this case, the conductivity within the cathodic compartment increased continuously from the onset (Figure S11). After 20 h, the precipitate within the cathodic compartment was filtered, collected and abbreviated T1. Electrolysis was restarted and continued for 72 h after which time the second fraction was filtered, collected and abbreviated T2. TGA, SEM, and EDX spectroscopy was conducted on both fractions (T1 and T2) and are depicted in Figure 5A–D. TGA results indicate the initial fraction (T1) obtained after 20 h in the absence of stirring is composed predominately of Mg(OH)_2_, confirmed further via EDX analysis (Ca 2.4 mol% vs. 47.2 mol% Mg Table S6). The TGA measurements from the second fraction T2 indicate the product contains Mg(OH)_2_, Ca(OH)_2,_ and CaCO_3_, again with CaCO_3_ resulting from carbonation of Ca(OH)_2_ during handling. The change in mass % for Mg(OH)_2_, Ca(OH)_2,_ and CaCO3 is 13.34%, 10.48%, and 3.55%, respectively. This corresponds to a ratio of 56.4 mol% Mg and 43.6 mol% Ca, in good agreement with EDX analysis of the surface (Ca 25.4 mol%, Mg 24.2 mol%), Table S7. SEM images of T1 (Figure 5C) indicate aggregates consisting of platelet‐like and flower‐shaped morphologies while SEM images of T2 (Figure 5D) indicate large hexagonal prism morphologies dispered within flower‐shaped microaggragates. Powder XRD from both fractions (T1 and T2) are displayed in Figure S12 and underline the TGA and EDX findings. Mixture agitation can be essential for effective carbonate dissolution, as the rate determining step of the dissolution process is dependent on the loss of CO_2_ from water which is achieved more rapidly through stirring [29].

Cumulatively, these mixtures separation techniques (time adjust or mixture agitation) suggest the precipitate Mg(OH)_2_ accumulates first followed by Ca(OH)_2_ precipitation. CaCO_3_ dissolves kinetically faster than MgCO_3_ at acidic to near‐neutral pH [30, 31], and Ca^2+^ ions are observed to permeate cation exchange membranes such as Nafion 115 more readily than Mg_2_^+^ ions due to Ca^2+^'s smaller hydrated radius [32]. Therefore, the observed product distribution of the collected phases (Mg(OH)_2_ precipiating first before Ca(OH)_2_ precipitation) can be attributed to the differences in precipitation kinetics of Mg(OH)_2_ compared with Ca(OH)_2_. Mg(OH)_2_ is observed to precipitate at less alkaline pH compared to that of Ca(OH)_2_ [33], although in these instances the pH is substantial enough to precipitate both compounds (vide supra). Previous works in understanding Ca(OH)_2_ and Mg(OH)_2_ nucleation kinetics indicate Mg(OH)_2_ is indeed expected to precipitate earlier in alkaline media due to faster nucleation rates compared to Ca(OH)_2_, with Ca(OH)_2_ requiring more units to conglomerate before precipitation occurs [34, 35]. This can be further conceptualized in the respectively crystal phase morphological structure (Figure 4C,D and 5C,D), as Ca(OH)_2_ growth requires a more controlled pathway leading to hexagonal prismatic crystal morphology. This product separation through controlling the extent of electrolysis time or mixture agitation holds practical application in product adjustment. Variation of such parameters within the electrolyzer can be utilized to steer product distribution and the resulting product ratio, leading to the possibility of fine‐tuning the alkali earth ion ratio in the resultant cement.

### 2.5 Implications in Cement Manufacturing

The transformation of the synthetic dolomite mixture into precursor phases suitable for cement manufacturing represents an important step toward utilizing nontraditional precursor materials in cement production. The electrochemical transformation of the synthetic dolomite mixture allows for the adjustment of alkali earth ions within the collected phases and underlines the utility of this process in tailoring such mixtures toward PC or Mg‐based cements. Ideally, a semi‐continuous electrochemical manufacturing technique could be conceptualized to first precipitate out the faster forming Mg(OH)_2_ species before Ca(OH)_2_ collection, thereby producing Mg‐ and Ca‐rich precursor streams for varied applications. Although this electrochemical approach displays promise in product phase adjustment, this laboratory scale system requires much further optimization specifically in aspects of cell design, mixture agitation, and heat integration opportunities to improve FE [36]. Moreover, although this laboratory scale system begins to demonstrate the possibility of refining such mixtures, the engineering challenges associated with up scaling the process remain considerable. This system would likely benefit from using flow cell electrolyzers with feed and bleed liquid and gas circulation [37, 38] or systems devoid of ion exchange membranes [39, 40, 41] avoiding issues in membrane passivation which are better suited for large‐scale electrolysis applications. Such systems can also allow for the continued recycle of electrolyte and prevent direct electrode–substrate contact, which can lead to catalyst degradation. However, such scalability challenges should not eclipse environmental benefits of such a process. Preliminary reports suggest that such large‐scale electrochemical cement production techniques can be equivocal in energy consumption in manufacturing [5, 11], yet allow for the use of renewable energy sources such as wind or solar energy to power such systems. Furthermore, if the O_2_ and H_2_ gases are collected effectively, such energy‐rich by‐products can be employed in sintering or other process. The authors note that energy metrics and comprehensive a techno‐economic analysis of the present system ideally requires a more sophisticated pilot‐scale electrolyzer system for validation. For a rigorous life‐cycle assessment and techno‐economic analysis of scalable electrified materials production and recycling, the reader is referred to Lu et al. [11]. Nevertheless, a screening‐level energy analysis of the current electrochemical system is within approximation. Basing the current metric of the FE of the electrolyzer (85.7% FE), the electrochemical dissolution of CaCO_3_‐MgCO_3_ mixtures consumed 1.05–1.37 kWh/kg, equivalent to 3.8–4.9 MJ/kg. This process generates both Ca(OH)_2_ for PC and Mg(OH)_2_ suitable for magnesia cements, with the latter requiring lower overall energy to produce as magnesia‐based cements are not clinkered [42]. The question remains if cementitious phases such as C‐S‐H or M‐S‐H can be generated directly from these hydroxide products without the need for clinkering or when mixed with amorphous silica. If successful, calcination—or in this case dihydroxylation to generate either CaO or MgO—might be circumvented entirely. In any case, if we consider clinkering still a prerequisite for traditional Ca‐based PC, when combined with clinkering energy, (2.95–3.40 MJ/kg), the total process would require 1.9–2.3 kWh/kg (6.7–8.3 MJ/kg) cement. At current renewable electricity price in Europe of €0.03−0.05/kWh [43], the dissolution energy therefore translates to approximately €31−68 per ton, combined with clinkering energy, the total process costs would be €56−116 per ton of PC. For comparison, conventional thermal cement production consumes on average 3.6–3.9 MJ/kg of thermal energy, with total fuel costs of approximately €10−15 per ton for thermal fuel [44, 45]. Economic viability will depend critically on electricity sourcing and, at present, this comparison excludes capital costs, and potential co‐product value of the generated H_2_, O_2_ gases from electrolysis, which require pilot‐scale data for reliable estimation. Importantly, however, Ellis et al. [5] suggest this apparent energy premium (72–131% above conventional) can be largely supplanted through heat recovery from electrochemically‐generated H_2_ and O_2_, reducing net supplemental energy requirements to ∼0.5 MJ/kg. The energy trade‐off must be evaluated against avoided process CO_2_ emissions (∼0.52 ton CO_2_/ton cement from calcination), and renewable electricity compatibility benefits unachievable via conventional thermal routes.

## 3 Conclusions

In summary, a mixture of CaCO_3_ and MgCO_3_ can be transformed into its respective hydroxide analogues (Ca(OH)_2_ and Mg(OH)_2_) via electrolysis. The reaction can be monitored through conductivity, temperature, and pH measurements, which elude to an approximate timeframe of the overall process. The electrolysis time scale and mechanical stirring affect product distribution of these hydroxide precursor phases, with Mg(OH)_2_ precipitating more rapidly than Ca(OH)_2_. These results suggest mixtures of calcium and magnesium carbonates can be transformed electrochemically into value‐added precursors for cement production and their product ratios adjusted. Further refinement of parameters governing electrochemical cement precursor production can allow for the utilization of such dolomite‐like source materials for PC and Mg‐based cement manufacturing.

## Supporting Information

Supporting information for this article is given via a link at the end of the document.

Link for Supporting information

Data for this article is available at Zonodo https://doi.org/10.5281/zenodo.17425418.

Additional supporting information can be found online in the Supporting Information section. **S** **upporting** **Fig. S1**: Linear sweep voltammetry (LSV) traces in the presence (blue trace) and absence (black trace) of substrate. The current is observed to increase at potentials >1.5 V vs. RHE. **Supporting** **Fig. S2**: Pulse Chronopotentiometry conducted at 100 mA with a voltage of approximately 2.1 V vs. Ag/AgCl resulting in an output power of ∼0.20 W at 30 min. Intervals with 2 min open circuit potential (OCP) between each pulse. **Supporting** **Fig. S3**: Picture of the anodic compartment of the electrochemical cell after 4.5 h of galvanostatic electrolysis. Arrows indicate where substrate build‐up has taken place away from the electrode. **Supporting** **Fig. S4**: Gas chromatograph of the gaseous products collected at the cathode during galvanostatic electrolysis at 100 mA.**Supporting** **Fig. S5**: XRD pattern of the hydroxide product mixture Ca(OH)2 and Mg(OH)2 heated at 650 °C for 2 h resulting in the respective oxide formation.**Supporting** **Fig. S6**: XRD pattern of the hydroxide product mixture Ca(OH)2 and Mg(OH)2 with 3:1 SiO2 heated at 900 °C for 5 h resulting trace amounts of the cementitious phase belite and the respective oxide formation.**Supporting** **Fig. S7**: SEM images of the hydroxide product mixture Ca(OH)2 and Mg(OH)2 3:1 SiO2 heated at 900 °C for 2 h indicating a glass‐like product within the mixture. A) 24000 x magnification B) 6000 x magnification.**Supporting** **Fig. S8**: Picture of the hydroxide product mixture Ca(OH)2 and Mg(OH)2 with 3:1 SiO2 heated at 1500 °C for 3 h forming a glass‐like ceramic material in a corundum crucible.**Supporting** **Fig. S9**: Ionic conductivity and temperature measurements of the reaction medium within the anodic compartment during chronopotentiometry at 100 mA in the presence of magnetic stirring, stopped after 2.5 h to collected Fraction F1 and 20 h to collect fraction F2.**Supporting** **Fig. S10**: XRD spectra of the collect fractions after controlled potential electrolysis at 100 mA. Fraction F1 collected after 2.5 h (A) Fraction F2 collected after 24 h (B).**Supporting** **Fig. S11**: Ionic conductivity and temperature measurements of the reaction medium within the anodic compartment during chronopotentiometry at 100 mA in the absence of magnetic stirring.**Supporting** **Fig. S12**: XRD spectra of the collect fractions after controlled potential electrolysis at 100 mA in the absence of magnetic stirring. Fraction T1 collected after 20 h (A) Fraction F2 collected after 72 h (B).**Supporting** **Table S1**: Gas chromatograph result analysis of gaseous products collected at the cathode during galvanostatic electrolysis at 100 mA.**Supporting** **Table S2**: Gaseous products collected at the cathode during galvanostatic electrolysis over mL intervals at 100 mA and calculated Faradaic efficiency (FE) at each volume.**Supporting** **Table S4**: EDX measurements conducted on 4 spots (01‐05) of the product mixture collected within the cathodic compartment after chronopotentiometry conducted at ∼0.20 W after 20 h.**Supporting** **Table S4**: EDX measurements conducted on 5 spots (01‐05) of the product collected at the cathode after chronopotentiometry conducted at ∼0.20 W after 2.5 h (Fraction F1) in the presence of magnetic stirring.**Supporting** **Table S5**: EDX measurements conducted on 5 spots (01‐05) of the product collected at the cathode after chronopotentiometry conducted at ∼0.20 W after 20 h (Fraction F2) in the presence of magnetic stirring.**Supporting** **Table S6**: EDX measurements conducted at 5 spots (01‐05) of the product collected at the cathode after chronopotentiometry conducted at ∼0.20 W after 20 h in the absence of magnetic stirring (T1). **Supporting** **Table S7**: EDX measurements conducted at 6 spots (01‐05) of the product collected at the cathode after chronopotentiometry conducted at ∼0.20 W after 72 h in the absence of magnetic stirring (T2).

## Funding

This work was supported by Deutsche Forschungsgemeinschaft (EXC 3115 project number 533767731, SFB/TRR280 Project number 417002380).

## Conflicts of Interest

The authors declare no conflicts of interest.

## Supporting information

Supplementary Material
