# Supplementary material for: Investigating Synthetic Dolomite Mixtures and Phase Adjustment Parameters in Electrochemical Cement Precursor Production
Source: Chemphyschem. 2026 Apr 22;27(8):e202500809. doi: 10.1002/cphc.202500809 (PMC13102320; doi:10.1002/cphc.202500809)
Supplement: Supplementary file 1 — Supplementary Material [file CPHC-27-e202500809-s001.pdf]

Investigating synthetic dolomite mixtures and phase adjustment parameters in electrochemical cement precursor production

Supplemental information

Anthony R. Ramuglia,[a,b*] Phong V. Ly,[a] Sophie Hurst-Fraunhofer,[a] Julius Scheel,[b] Kelly Henze,[c] Thomas Köberle,[b] Volodymyr Bon,[d] Stefan Kaskel,[d] Viktor Mechtcherine,[b] Thomas Matschei,[e] Marco Liebscher[b*] and Inez M. Weidinger[a*]

[a.] *Chair of Electrochemistry, Faculty of Chemistry and Food Chemistry*
Technische Universität Dresden
Zellescher Weg 19, 01069 Dresden (Germany)
E-mail: anthony.ramuglia@tu-dresden.de Inez.Weidinger@tu-dresden.de
[b.] *Institute of Construction Materials*
Technische Universität Dresden
Georg-Schumann-Straße 7, 01187 Dresden (Germany)
E-mail: marco.liebscher@tu-dresden.de
[c.] *Chair of Physical Chemistry, Faculty of Chemistry and Food Chemistry*
Technische Universität Dresden
Zellescher Weg 19, 01069 Dresden (Germany)
[d.] *Chair of Inorganic Chemistry I, Faculty of Chemistry and Food Chemistry*
Technische Universität Dresden
Bergstrasse 66, 01069 Dresden (Germany)
[e.] *Institute of Building materials research and Chair of Building Materials*
RWTH Aachen University
Schinkelstrasse 3, 52062 Aachen (Germany)

Contents

Experimental 3
Electrochemistry 4
Electrolyzer efficiency 5
Product analysis of hydroxide mixtures 7
Product analysis after heat treatment 7
Electrolysis time adjustment 9
Mixture agitation adjustment 11

Experimental

Electrolysis experiments were conducted using a two compartment cell. Unless otherwise stated, all electrochemical measurements were performed in a two compartment "H-cell" containing a total volume of 140 mL, consisting of 70 mL of supporting electrolyte in each compartment. The two compartments were partitioned with a Nafion 115 cation exchange membrane. The membrane was activated through first treatment in 3 wt % H_2O_2 for one hour at 80 °C followed by treatment in deionized water at 80 °C for one hour, subsequent treatment in 0.5 M H_2SO_4 at 80 °C for one hour and finally rinsed and stored in deionized water at room temperature. A Pt mesh electrode on Ti was utilized for both the cathode and anode, both measuring approximately 30 cm x 35 cm. An Ag/AgCl electrode in 3 M KCl solution separated by a glass frit constituted the reference electrode. Milli Q deionized water with a conductance of 0.055 µS was used for all experiments. 0.5 M KNO_3 (Grüssing GmbH) was used as the electrolyte in all experiments.

For electrolysis all experiments a mixture of 0.5 g of $CaCO_3$ (5.0 x 10^{-3} mol) (Grüssing GmbH) and 0.5 g $4MgCO_3\ Mg(OH)_2{\cdot}5H_2O$ (4.1 x 10^{-3} mol $MgCO_3$) (Carl-Roth GmbH) were used as received and functioned as the starting material. The hydroxide products were collected via vacuum filtration, washed with several portions of deionized water, left in the fume hood to an oven at 85 °C for approximately 4 hrs before being stored in a in desiccator.

Electrochemical experiments were conducted using an Ivium Vertex One EIS potentiostat with Ivium electrochemical software and Biologic SP-300 potentiostat with EC-Lab software

pH, temperature and conductivity measurements were conducted on an APERA820 pH and conductivity meter. For pH measurements, the electrolysis was halted every 30 min and the electrodes were disconnected before an accurate reading of the system could be taken. Conductivity measurements were taken every 30 min during electrolysis, with the applied current pulsed from to open circuit potential (OCP) every 30 min and conductivity measurements taken every 31 min to allow for one minute equilibration time.

Gas Chromatograph measurements were conducted using a Thermo Fischer 1300 Gas Chromatograph with TG-Bond Q and TG-Bond Msieve 5A connected in series.

XRD measurements were performed on a Bruker 2D phaser equipped with Cu-Kα_1 with λ = 1.54059 Å radiation. Samples were dispersed in ethanol, drop-cast on a Si wafer and dried in ambient air. Additional experiments were performed on an Empyrean (PANALYTICAL) powder X-ray diffractometer (3rd Generation, alpha-1 system), equipped with Cu-Kα_1 radiation (primary "Johansson type" monochromator λ = 1.54059 Å), motorized anti-scatter and receiving slits and PIXcel 1D detector. The samples were prepared using the background-free Si sample holder. In all cases PXRD patterns were collected in reflection geometry using ω-2θ scans in the 2θ range from 5 - 90° with 0.017° steps and 100 s per step.

SEM images were taken on a ESEM Quanta FEG250 (FEI) with connected EDX QUANTAX 400 (Bruker).

EDX measurements were performed on the respective powder at magnification 2000x (frame: 200 x 140 µm) with 20kV and spot size 4.0 µm, with 5 measurements carried out on each sample.

TGA was carried out using an STA 409 cell device from Netzsch, Germany, under an helium atmosphere, operated with a heating rate of 10 K/min, ranging from 20 to 1000 °C, and 60 ml/min gas flow. Additional TGA measurements, specifically Figure 4, T1 was carried out using a Mettler Toledo TGA/DSC 1 STAR System, under a nitrogen atmosphere operated with a heating rate of 15 K/min, ranging from 30 to 1000 °C, and 250 ml/min gas flow.

Electrochemistry

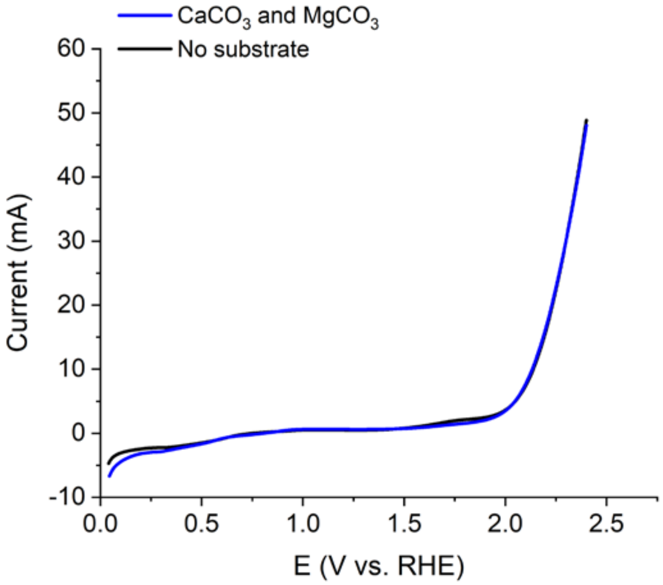

Figure S1. Linear sweep voltammetry (LSV) traces in the presence (blue trace) and absence (black trace) of substrate. The current is observed to increase at potentials >1.5 V vs. RHE

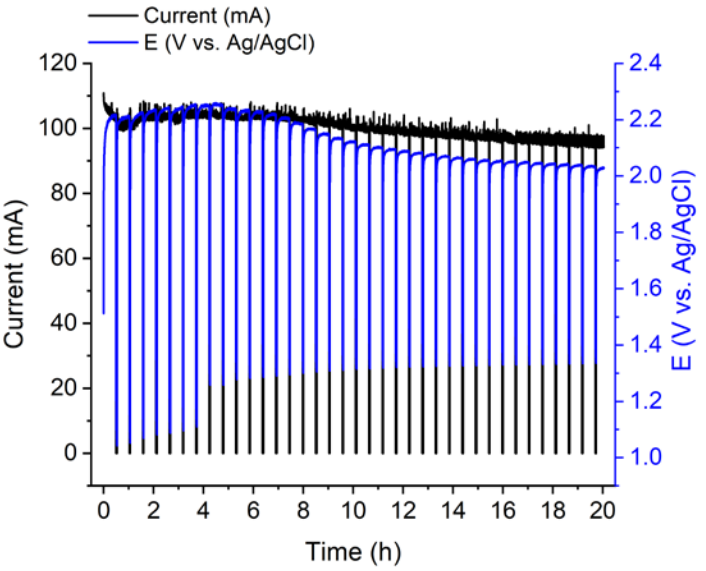

Figure S2. Pulse Chronopotentiometry conducted at 100 mA with a voltage of approximately 2.1 V vs. Ag/AgCl resulting in an output power of ~0.20 W at 30 min. Intervals with 2 min open circuit potential (OCP) between each pulse.

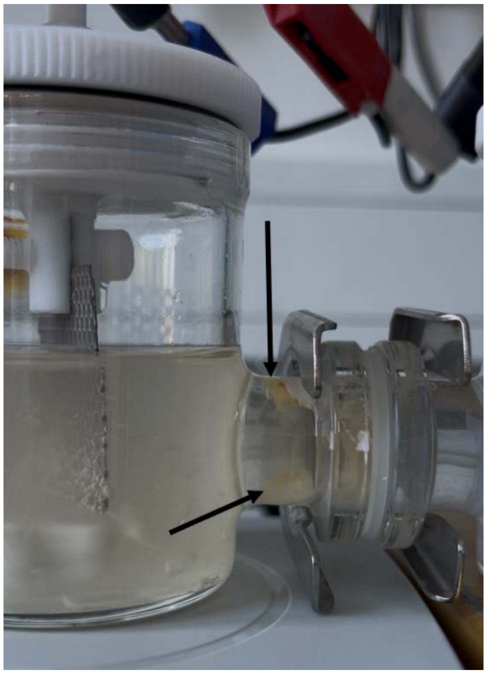

Figure S3. Picture of the anodic compartment of the electrochemical cell after 4.5 h of galvanostatic electrolysis. Arrows indicate where substrate build-up has taken place away from the electrode.

Electrolyzer efficiency

At 20 °C, H_2 was collected (mL) over time at the cathode during galvanostatic electrolysis at 100 mA. Gas chromatography indicates the collect gas contain 81 % H_2.

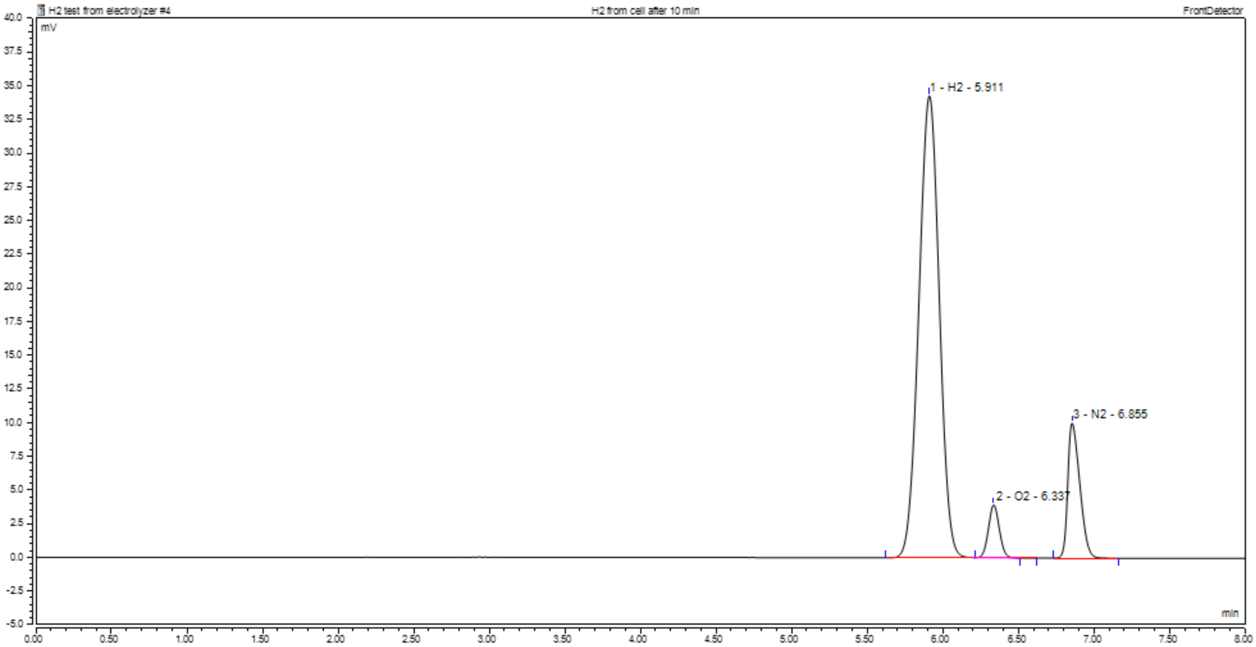

Figure S4. Gas chromatograph of the gaseous products collected at the cathode during galvanostatic electrolysis at 100 mA

Table S1. Gas chromatograph result analysis of gaseous products collected at the cathode during galvanostatic electrolysis at 100 mA.

Peak	Peak Name	Ret.Time	Rel.Area	Area	Height
No.		min	%	mV*min	mV
FrontDetector	FrontDetector	FrontDetector	FrontDetector	FrontDetector	FrontDetector
1	H2	5.911	81.11	5.3689	34.31
2	O2	6.337	4.95	0.3277	3.92
3	N2	6.855	13.94	0.923	10.03
Maximum			81.11	5.3689	34.31
Minimum			4.95	0.3277	3.92
Sum			100	6.6196	48.26

Table S2. Gaseous products collected at the cathode during galvanostatic electrolysis over mL intervals at 100 mA and calculated Faradaic efficiency (FE) at each volume.

Volume (mL)	Time (s)	Effective H_2 Vol (mL)	Moles H_2	Q Theorectical (C)	Q Experimental (C)	FE (%)
2	150	1.62	6.74E-05	13	15	86.6
3	225	2.43	1.01E-04	19.5	22.5	86.6
4	306	3.24	1.35E-04	26.2	30.6	85.6
5	385	4.05	1.69E-04	32.6	38.5	84.7
6	465	4.86	2.03E-04	39.2	46.5	84.4
7	540	5.67	2.37E-04	45.8	54	84.8
8	607	6.48	2.71E-04	52.3	60.7	86.2
9	675	7.29	3.05E-04	58.9	67.5	87.3
10	770	8.10	3.39E-04	65.5	77	85.1

Average FE = 85.7 %

Product analysis of hydroxide mixtures

Table S4. EDX measurements conducted on 4 spots (01-05) of the product mixture collected within the cathodic compartment after chronopotentiometry conducted at ~0.20 W after 20 h.

Spectrum (mol %, normalized)	Mg	Al	Si	K	Ca	S	O
Cathodic filtrate_CaMg_1-1_100mA_01	23.5	0.0	0.1	0.1	26.4	0.0	49.9
Cathodic filtrate_CaMg_1-1_100mA_02	23.4	0.0	0.0	0.1	26.4	0.0	50.1
Cathodic filtrate_CaMg_1-1_100mA_03	25.2	0.1	0.1	0.0	24.5	0.0	50.1
Cathodic filtrate_CaMg_1-1_100mA_04	25.2	0.1	0.1	0.1	24.3	0.1	50.1
Cathodic filtrate_CaMg_1-1_100mA_05	27.2	0.0	0.1	0.1	22.6	0.0	50.0
mean	24.9	0.0	0.1	0.1	24.8	0.0	50.1
standard deviation	1.4	0.0	0.0	0.0	1.4	0.1	0.1

Product analysis after heat treatment

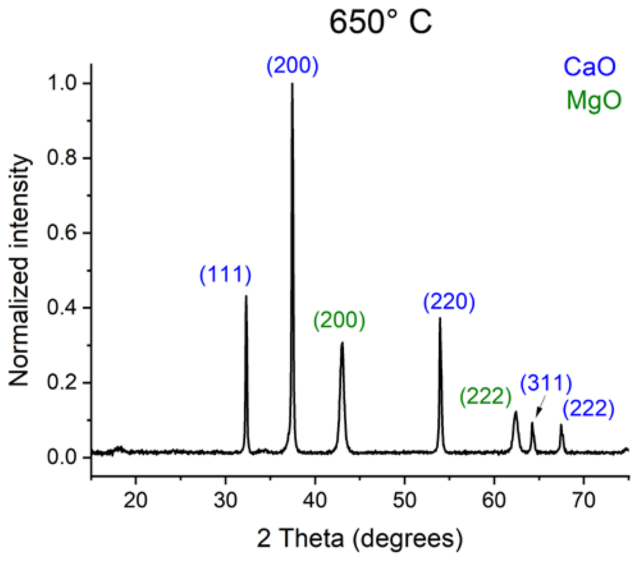

Figure S5. XRD pattern of the hydroxide product mixture $Ca(OH)_2$ and $Mg(OH)_2$ heated at 650 °C for 2 h resulting in the respective oxide formation.

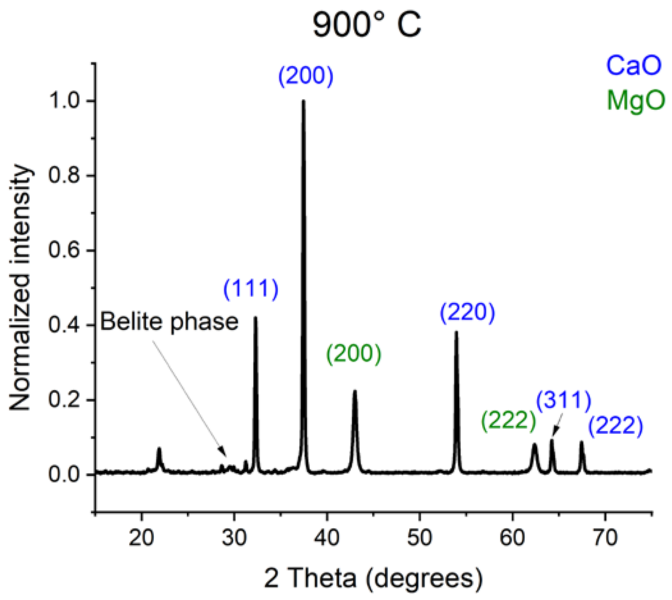

Figure S6. XRD pattern of the hydroxide product mixture $Ca(OH)_2$ and $Mg(OH)_2$ with 3:1 SiO_2 heated at 900 °C for 5 h resulting trace amounts of the cementitious phase belite and the respective oxide formation.

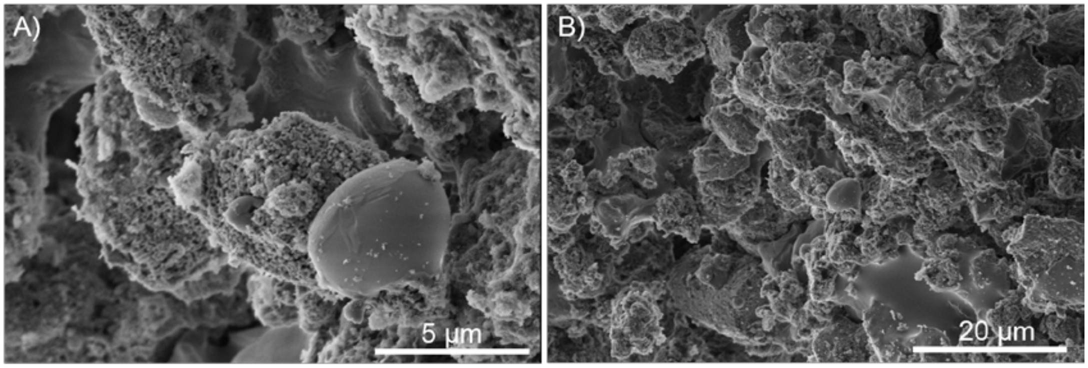

Figure S7. SEM images of the hydroxide product mixture $Ca(OH)_2$ and $Mg(OH)_2$ 3:1 SiO_2 heated at 900 °C for 2 h indicating a glass-like product within the mixture. A) 24000 x magnification B) 6000 x magnification

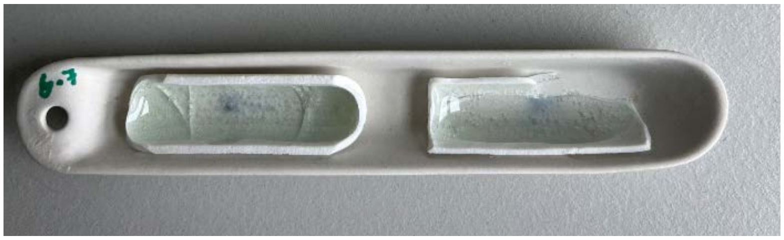

Figure S8. Picture of the hydroxide product mixture $Ca(OH)_2$ and $Mg(OH)_2$ with 3:1 SiO_2 heated at 1500 °C for 3 h forming a glass-like ceramic material in a corundum crucible.

Electrolysis time adjustment

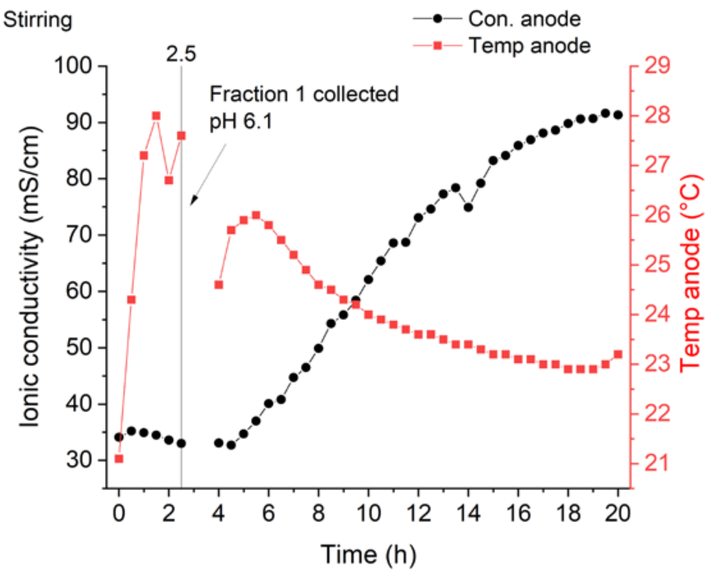

Figure S9. Ionic conductivity and temperature measurements of the reaction medium within the anodic compartment during chronopotentiometry at 100 mA in the presence of magnetic stirring, stopped after 2.5 h to collected Fraction F1 and 20 h to collect fraction F2.

Table S4. EDX measurements conducted on 5 spots (01-05) of the product collected at the cathode after chronopotentiometry conducted at ~0.20 W after 2.5 h (Fraction F1) in the presence of magnetic stirring.

Spectrum (mol %, normalized)	K	Ca	Mg	O
Cathodic filtrate_F1_100mA_stir_01	0.0	2.5	47.5	50.0
Cathodic filtrate_F1_100mA_stir_02	0.1	2.6	47.4	50.0
Cathodic filtrate_F1_100mA_stir_03	0.1	1.8	48.1	50.0
Cathodic filtrate_F1_100mA_stir_05	0.1	1.6	48.4	49.9
Cathodic filtrate_F1_100mA_stir_06	0.1	3.0	47.0	49.9
mean	0.1	2.3	47.7	50.0
Standard deviation	0.0	0.5	0.5	0.0

Table S5. EDX measurements conducted on 5 spots (01-05) of the product collected at the cathode after chronopotentiometry conducted at ~0.20 W after 20 h (Fraction F2) in the presence of magnetic stirring.

Spectrum (mol %, normalized)	K	Ca	Mg	O
Cathodic filtrate_F2__100mA_stir_01	2.5	6.1	42.0	49.4
Cathodic filtrate_F2__100mA_stir_02	6.1	8.6	36.6	48.4
Cathodic filtrate_F2__100mA_stir_03	1.5	9.8	39.0	49.6
Cathodic filtrate_F2__100mA_stir_04	0.9	11.3	38.1	49.8
Cathodic filtrate_F2__100mA_stir_05	1.4	14.6	34.3	49.6
mean	2.5	10.1	38.0	49.4
standard deviation	1.9	2.8	2.5	0.5

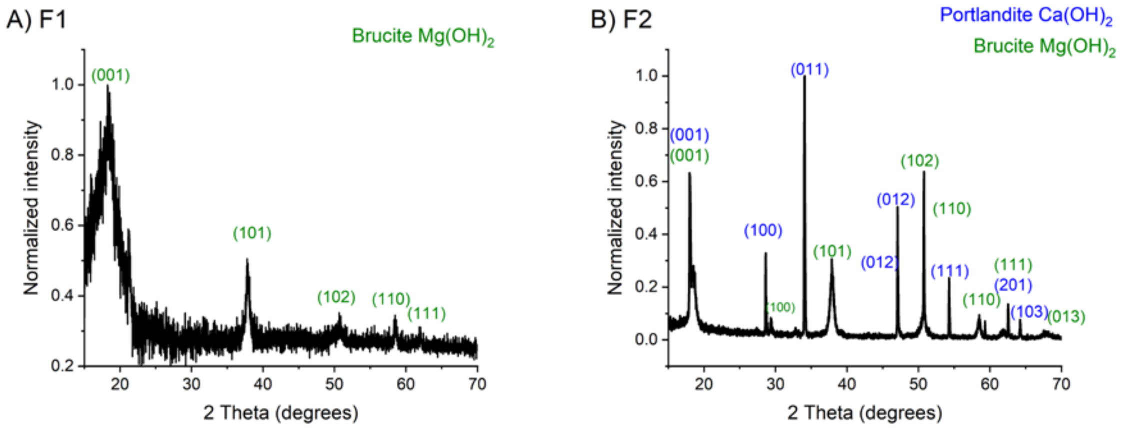

Figure S10. XRD spectra of the collect fractions after controlled potential electrolysis at 100 mA. Fraction F1 collected after 2.5 h (A) Fraction F2 collected after 24 h (B).

Mixture agitation adjustment

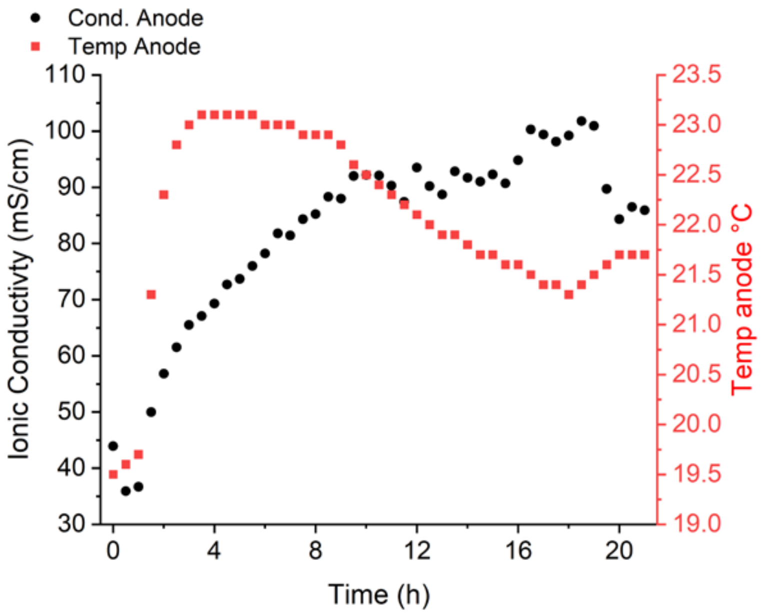

Figure S11. Ionic conductivity and temperature measurements of the reaction medium within the anodic compartment during chronopotentiometry at 100 mA in the absence of magnetic stirring.

Table S6. EDX measurements conducted at 5 spots (01-05) of the product collected at the cathode after chronopotentiometry conducted at ~0.20 W after 20 h in the absence of magnetic stirring (T1).

Spectrum (mol %, normalized)	Na	Mg	Ca	O
Cathodic Filtrate _T1_100mA_ no stir_01	0.3	46.4	3.3	50.0
Cathodic Filtrate _T1_100mA_ no stir_02	0.2	48.0	1.7	50.0
Cathodic Filtrate _T1_100mA_ no stir_03	0.0	47.1	2.6	50.1
Cathodic Filtrate _T1_100mA_ no stir_04	0.3	47.7	1.9	50.0
Cathodic Filtrate _T2_100mA_ no stir_05	0.0	46.9	2.7	50.1
mean	0.1	47.2	2.4	50.1
standard deviation	0.1	0.5	0.5	0.1

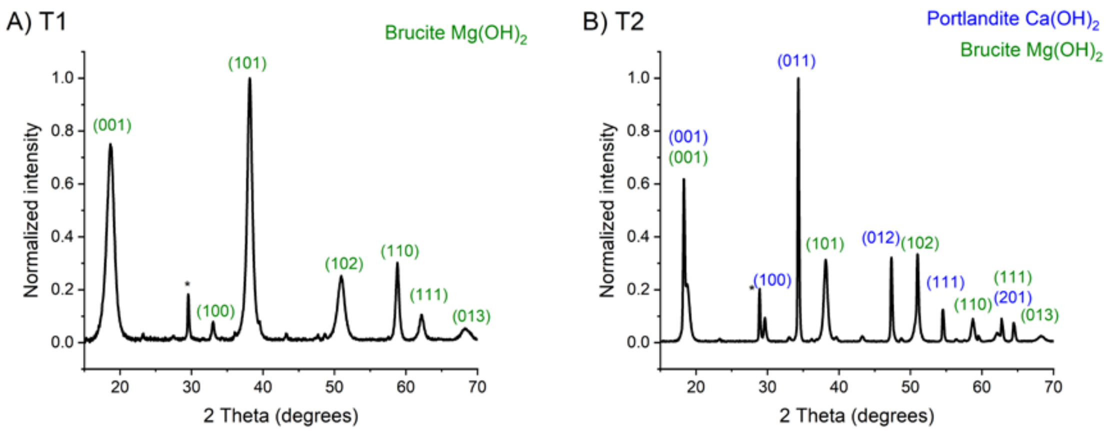

Figure S12. XRD spectra of the collect fractions after controlled potential electrolysis at 100 mA in the absence of magnetic stirring. Fraction T1 collected after 20 h (A) Fraction F2 collected after 72 h (B).

Table S6. EDX measurements conducted at 6 spots (01-05) of the product collected at the cathode after chronopotentiometry conducted at ~0.20 W after 72 h in the absence of magnetic stirring (T2).

Spectrum	Ca	Mg	Si	K	O
Cathodic Filtrate _T2_100mA_ no stir_01	27.31	22.31	0.12	0.26	50
Cathodic Filtrate _T2_100mA_ no stir_02	28.52	21.16	0.08	0.26	49.98
Cathodic Filtrate _T2_100mA_ no stir_03	28.27	21.47	0.04	0.26	49.96
Cathodic Filtrate _T2_100mA_ no stir_04	29.24	20.44	0.08	0.26	49.98
Cathodic Filtrate _T2_100mA_ no stir_05	21	28.52	0.04	0.55	49.88
Cathodic Filtrate _T2_100mA_ no stir_06	18.93	30.49	0.12	0.54	49.92
Mean	25.55	24.07	0.08	0.36	49.95
Sigma	4.41	4.3	0.04	0.15	0.04
